# Supplementary figures and images for: Identifying and validating ITGB2 and HNRNPAB as diagnostic biomarkers in chronic obstructive pulmonary disease using bioinformatics and Integrated Machine Learning Methods
Source: PLoS One. 2026 May 21;21(5):e0349338. doi: 10.1371/journal.pone.0349338 (PMC13193535; doi:10.1371/journal.pone.0349338)

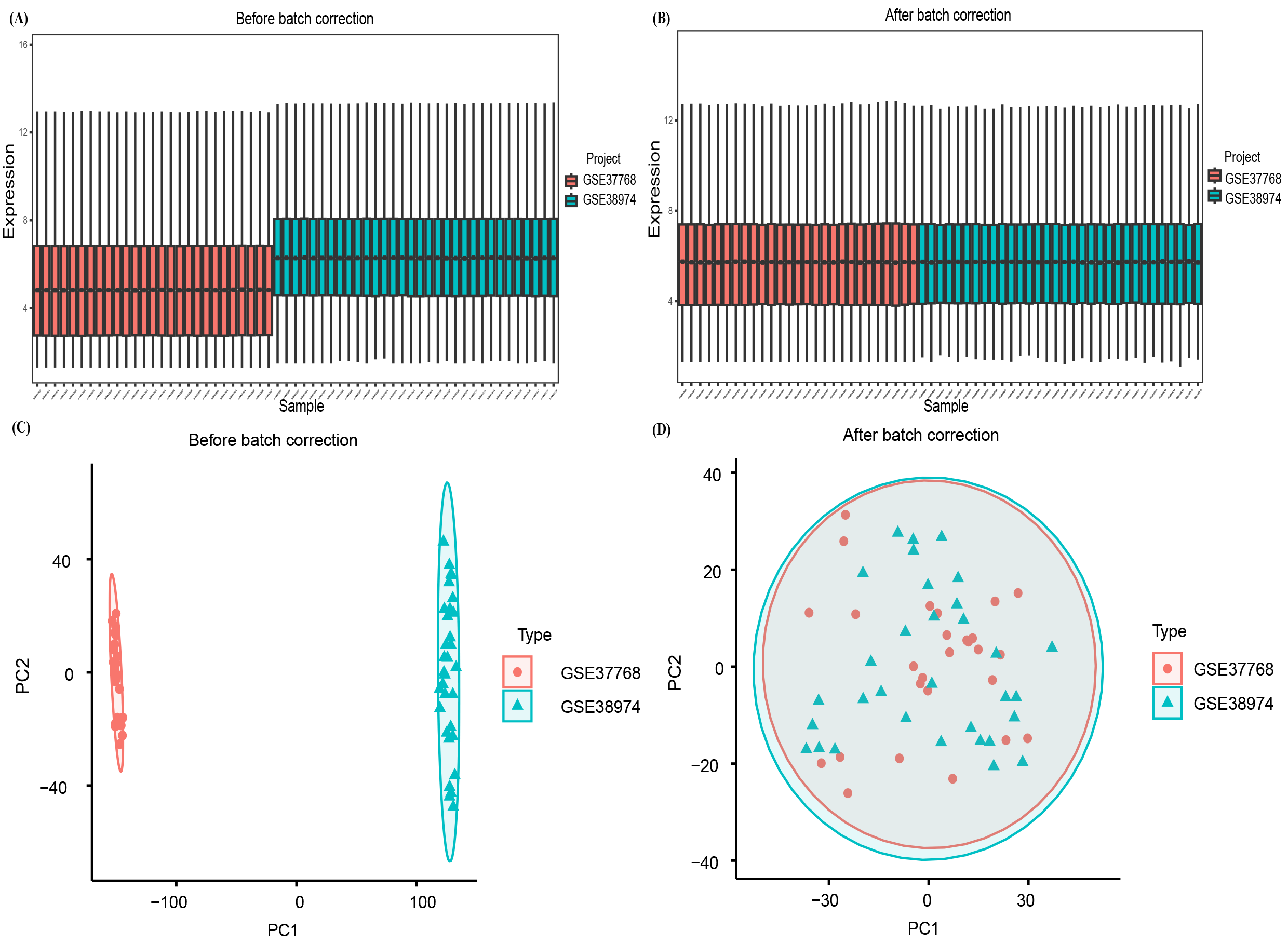

Supplement: S1 Fig — (TIF) [file pone.0349338.s002.tif]

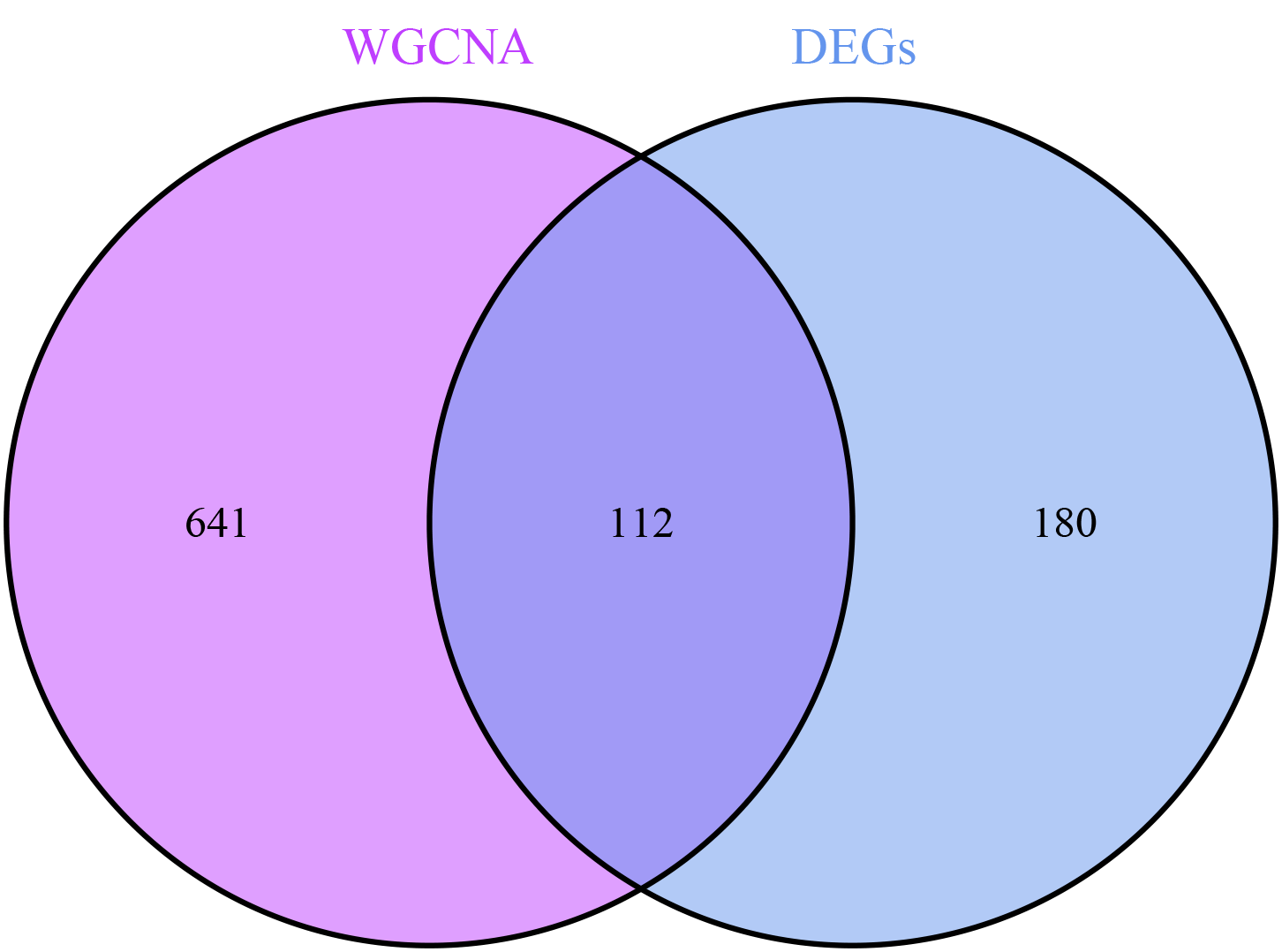

Supplement: S2 Fig — (TIF) [file pone.0349338.s003.tif]
