# Supplementary material for: Identifying and validating ITGB2 and HNRNPAB as diagnostic biomarkers in chronic obstructive pulmonary disease using bioinformatics and Integrated Machine Learning Methods
Source: PLoS One. 2026 May 21;21(5):e0349338. doi: 10.1371/journal.pone.0349338 (PMC13193535; doi:10.1371/journal.pone.0349338)
Supplement: S1 Table — (DOCX) [file pone.0349338.s004.docx]

**S1 Table** Relevant functions of 12 COPD diagnostic molecular markers screened by machine learning integrated framework.

| **Gene** | **Full name** | **Gene Function** |
| --- | --- | --- |
| *CD300C* | CD300 molecule-like family member C | Regulates immune processes, specifically expressed on CD56bright Natural Killer Cells, and enhances effector functions of these cells upon activation. |
| *GNG13* | Guanine nucleotide binding protein, gamma 13 | Part of the G protein family, involved in signal transduction. |
| *HNRNPAB* | Heterogeneous nuclear ribonucleoprotein A/B | Involved in RNA processing, transport, and translation.Studies showing the integration of lung tissue gene expression with genome-wide SNP genotyping found that *HNRNPAB* is associated with COPD, and this gene has been shown to induce EMT, which in turn causes airway related diseases. |
| *ITGB2* | Integrin, beta 2 | Encodes β2 integrin, important for cell adhesion and signaling. *ITGB2* expression of CD4-delivered small extracellular vesicles may be associated with inflammation and cell proliferation in COPD. |
| *LDB2* | LIM domain binding 2 | Encodes a protein with LIM domains, involved in various cellular processes. |
| *LTC4S* | Leukotriene C4 synthase | Encodes an enzyme involved in inflammation and immune responses, associated with asthma and other respiratory conditions. *LTC4S* participated in the construction of emphysema model mice and played a role in quantifying the compliance of diseases related to airway changes. |
| *MCOLN3* | Mucolipin 3 | Involved in vesicle transport and intracellular trafficking. *MCOLN3* is expressed in lung-running macrophages, regulates the expression of *MMP-12*, mediates the development of lung injury and emphysema, and then affects the progression of COPD. |
| *NAIP* | NLR family, apoptosis inhibitors protein | Part of the NLR family, involved in inflammasome activation and inhibition of apoptosis. Plays a role in host defense against bacterial infections and may be involved in the immune response in COPD. |
| *NOV* | Nephroblastoma overexpressed gene | Encoded protein is involved in cell adhesion and migration. |
| *PDAP1* | Pdap1 protein | Function not completely clear, may be involved in cellular metabolism and energy production. Previous studies have shown that *PDAP1* is significantly up-regulated in lung tissue and bronchial epithelial cells (BEAS-2B) of smoke-induced COPD mice treated with cigarette smoke extract, and is involved in the diagnosis and evaluation of COPD. |
| *S100B* | S100 calcium binding protein B | Involved in calcium binding and cellular signaling. Previous studies have shown that *S100B* is significantly related to B cell marker genes and participates in immune response, and is specifically expressed in COPD brain injury. |
| *SMAD7* | SMAD family member 7 | Involved in TGF-beta signaling pathway. *SMAD7* is involved in the regulation of inflammatory response and tissue remodeling in COPD by antagonizing TGF-β, leading to airway remodeling and exacerbation of disease symptoms. In addition, *SMAD7* influences the balance between pro-inflammatory and anti-inflammatory cytokines in the lung microenvironment mediating COPD inflammation. |
