# Supplementary material for: Identifying and validating ITGB2 and HNRNPAB as diagnostic biomarkers in chronic obstructive pulmonary disease using bioinformatics and Integrated Machine Learning Methods
Source: PLoS One. 2026 May 21;21(5):e0349338. doi: 10.1371/journal.pone.0349338 (PMC13193535; doi:10.1371/journal.pone.0349338)
Supplement: Attachment — Submitted filename: Response to Reviewers.docx [file pone.0349338.s006.docx]

List of Responses

Dear Miquel Vall-llosera Camps and Reviewer:

Thank you for your letter and for the reviewers’ comments concerning our manuscript entitled “Identifying and Validating ITGB2 and HNRNPAB as diagnostic biomarkers in chronic obstructive pulmonary disease Using Bioinformatics and Integrated Machine Learning Methods”. (Manuscript Number: PONE-D-25-52871). Those comments are all valuable and very helpful for revising and improving our paper, as well as the important guiding significance to our research. We have studied comments carefully and have made corrections which we hope meet with approval. Revised portions are marked with a yellow background in the revised version. The main corrections in the paper and the responds to the reviewer’s comments are as flowing:

Responses to reviewer’s comments: (original comments by reviewers are in blue color). Furthermore, we have added the legend explanations as per the requirements, which are located at the bottom of the manuscript (lines 817 to 866).

Reviewer #1:

1. Response to comment: The manuscript by Zhang et al.” Identifying and Validating ITGB2 and HNRNPAB as diagnostic biomarkers in chronic obstructive pulmonary disease Using Bioinformatics and Integrated Machine Learning Methods" identifies COPD biomarkers from multiple gene expression datasets deposited in the GEO database, by combining several bioinformatics approaches including WGCNA, immune infiltration profiling, transcription factor and regulatory network analysis, enrichment analyses, MR, GSVA, PCR validation, and integrated machine-learning methods. Two of the identified biomarkers, ITGB2 and HNRNPAB, were further validated through PCR, IHC and IF experiments in a mouse model.
The experiments are well designed, appropriately analyzed, and the methodology is clearly described throughout the manuscript. The results are of interest to the field and, therefore, I recommend publication after minor revisions aimed at further improving the clarity and completeness of the work.
Minor comments:
1. Methods and Materials (line 100):
The sentence “…and GSE1650 consists of 18 COPD patient samples and 12 healthy control samples [17]…” is incomplete. Please clarify for what purpose this dataset was used in the study.

Response: Thank you very much for the Reviewer’s comments. We have already provided supplementary explanations for the original text. In this study, we first conducted difference analysis and WGCNA analysis on the GSE37768 and GSE38974 datasets merged by the sva algorithm. The intersected differential gene inputs were further analyzed by 113 machine learning models. The GSE212331, GSE148004 and GSE1650 datasets were used as independent queues to verify the stability of the above-mentioned machine learning models.

2. Response to comment: Identification of DEGs in COPD (lines 109–110):
The authors state that genes were defined as DEGs if they met the criteria of |log Fold Change| ≥ 0.585 and adjusted p-value < 0.05. Why was the threshold set at 0.585 specifically, rather than 0.586, 0.584, or a more conventional 0.5? Please provide a justification for this choice.

Response: Special thanks to you for your exact and professional comments. We set the log2 FoldChange to 0.585 based on the principle of logarithmic transformation. The location of the difference multiple is "gene expression level of the treatment group/gene expression level of the control group" (up-regulated genes) or "gene expression level of the control group/gene expression level of the treatment group" (down-regulated genes). Since the expression levels of RNA-seq data (such as FPKM and TPM) usually follow a lognormal distribution, logarithmic transformation to the base 2 (log2 (FC)) is commonly used in bioinformatics analysis to make the data meet the normality hypothesis and facilitate subsequent statistical tests. When FC=1.5, the corresponding log2 (FC)=log2 (1.5)≈0.58496. To simplify the calculation and ensure the uniformity of the threshold, this study rounded it to 0.585. In previous studies on respiratory diseases, the setting of this threshold was also used by other researchers [1]. This conversion logic is a standard operation for screening differentially expressed genes in bioinformatics analysis, ensuring the mathematical rigor of the threshold setting. Thank you for your review. We have revised the original text, which ensures the accuracy of the conclusion.

Reference:

1.	Wu Z, Chen H, Ke S, Mo L, Qiu M, Zhu G, et al. Identifying potential biomarkers of idiopathic pulmonary fibrosis through machine learning analysis. Sci Rep. 2023;13(1):16559. doi: 10.1038/s41598-023-43834-z. PubMed PMID: 37783761; PubMed Central PMCID: PMCQ2.

3. Response to comment: Discussion section:
The results obtained from the validation of ITGB2 and HNRNPAB in the mouse model should be revisited and discussed in this section, clearly justifying their purpose and contribution to the interpretation and validation of the findings.

Response: Thank you sincerely for your meticulous review and highly constructive suggestions. Based on your suggestions, we have made extensive revisions to the discussion section of the paper. We have systematically analyzed a variety of biological and technical reasons that may lead to inconsistent expression trends at mRNA and protein levels. We emphasize that this discovery has not weakened the value of ITGB2 and HNRNPAB as important biomarkers for COPD. On the contrary, it suggests that in the complex pathological environment of COPD, gene expression regulation has multi-level and dynamic characteristics. Computational biology screening has helped us identify these two genes strongly associated with diseases, and the "contradictions" revealed by wet experimental verification precisely point to the possible existence of novel biological mechanisms (such as active protein turnover or translation inhibition), which in itself is a valuable discovery. During the revision, we also candidly admitted the limitations of the current research, that is, the initial verification failed to fully unify the expression level. We have taken this as the starting point for future research. At the end of the discussion, we proposed several hypotheses and experimental directions that can be further verified (such as detecting protein degradation pathways and conducting functional recovery experiments, etc.) to promote subsequent exploration in this field.

We believe that the revised discussion section not only directly addresses your concerns but also enhances the scientific depth of the paper, transforming seemingly contradictory data into an opportunity to tell a richer biological story.

Thank you again for your valuable suggestions. We hope our revisions can earn your approval of the paper.

4. Response to comment:

In addition, the sentence “The transcriptional factors FOXC1 and NFIC play an important role in the regulation of COPD” (lines 446–447) should be better integrated with the other proteins/pathways identified and discussed in the manuscript.

Response: We thank the reviewer for the critical suggestion. In response, we have thoroughly revised the discussion to integrate FOXC1 and NFIC into our molecular narrative. We moved beyond stating their“important role”to highlighting a concrete regulatory relationship predicted by our network analysis: NFIC directly targets HNRNPAB. This axis is presented as a central piece connecting upstream regulation (TF network), core biomarkers (HNRNPAB’s prioritization by MR), and downstream pathology. This creates a cohesive story from discovery to mechanistic insight.

We believe this revision directly addresses your comment by deeply embedding the transcription factors within a specific and plausible biological framework derived from our own data.
