# Supplementary material for: Identifying and validating ITGB2 and HNRNPAB as diagnostic biomarkers in chronic obstructive pulmonary disease using bioinformatics and Integrated Machine Learning Methods
Source: PLoS One. 2026 May 21;21(5):e0349338. doi: 10.1371/journal.pone.0349338 (PMC13193535; doi:10.1371/journal.pone.0349338)
Supplement: Attachment — Submitted filename: Response_to_Reviewers_auresp_2.docx [file pone.0349338.s007.docx]

List of Responses

Dear Tomasz W. Kaminski and Reviewer:

Thank you for your letter and for the reviewers’ comments concerning our manuscript entitled “Identifying and Validating ITGB2 and HNRNPAB as diagnostic biomarkers in chronic obstructive pulmonary disease Using Bioinformatics and Integrated Machine Learning Methods”. (Manuscript Number: PONE-D-25-52871). Those comments are all valuable and very helpful for revising and improving our paper, as well as the important guiding significance to our research. We have studied comments carefully and have made corrections which we hope meet with approval. Revised portions are marked with a yellow background in the revised version. The main corrections in the paper and the responds to the reviewer’s comments are as flowing:

Responses to reviewer’s comments: (original comments by reviewers are in blue color).

Reviewer #2:

Response to comment: I understood the authors' purpose and motivation for this study. Unfortunately, however, methods of some analyses and experiments were totally unclear or insufficiently described, making the results and conclusion of this study totally unconvincing and not supported with valid evidence. In particular, I do not think that it is reasonable and well-supported to focus only on ITGB2 and HNRNPAB genes from the analysis results.
At least the authors should have concretely explained (1) the source of SNPs affecting protein expression levels; (2) rationale of “TF-gene regulatory network" (based on TF binding sites? or co-expression?); (3) excuse for arbitrarily selecting the IVW method in the MR analysis while some other methods returned insignificant results; (4) how to establish the “COPD mouse model” and its validity; and (5) whether antigen retrieval in immunohistochemistry on the model tissue was surely succeeded.

In addition, there are many typos and mis-description in this manuscript which probably cause misunderstanding and misleading: always spell the two "key" genes at least correctly; legends of Fig. 3D and Fig. 6 do not correspond to the contents of those figures.

Response:

(1) We sincerely appreciate the reviewer’s professional and rigorous comment. We have supplemented the exact source of the protein expression quantitative trait locus (pQTL) SNPs used in the two-sample Mendelian randomization (MR) analysis as follows: The SNPs used as instrumental variables for ITGB2 and HNRNPAB protein expression were obtained from large-scale human plasma proteome-wide association studies (pQTLs) publicly available in the UK Biobank and the deCODE genetics proteome database, which are the most commonly used and authoritative pQTL resources in MR studies. Specifically, we obtained genome-wide significant protein quantitative trait loci (pQTLs) for ITGB2 and HNRNPAB using a p < 5×10⁻⁸ threshold. These SNPs have been experimentally verified to be strongly associated with circulating protein levels in large human population cohorts. We only included independent SNPs after linkage disequilibrium (LD) clumping (r² < 0.01, window size = 10,000 kb). We have added this description in the Methods (Section 2.9) of the revised manuscript to clarify the source, screening criteria, and quality control of the instrumental SNPs, ensuring full transparency and reproducibility.

(2) We sincerely appreciate the professional and insightful comment.

The rationale of the “TF‑gene regulatory network” in our study is based on transcription factor (TF) binding sites and experimentally validated regulatory relationships, rather than simple co‑expression.

(3) We sincerely appreciate the professional and insightful comment regarding the methodological choice in the Mendelian randomization (MR) analysis. The IVW method was used as the primary MR method because it is the most standard, powerful, and widely recommended approach for causal inference in Mendelian randomization. Other methods (e.g., weighted median, MR-Egger) have lower statistical power and different model assumptions, which may lead to insignificant results even when a true causal effect exists. The selection was not arbitrary but consistent with standard MR analytical practice.

(4) The mice were randomly divided into 2 groups: the control group and the COPD model group. The model group was exposed to cigarette smoke for 2 hours daily (30 cigarettes each time), 6 days a week for a total of 8 weeks. On days 1, 14, and 28, they received intravenous infusion of LPS (50 μg per mouse). Compared with the control group, the COPD mouse model needed to meet the following core indicators to be considered a successful model establishment [1-3]: ① Behavioral characteristics: The fur becomes yellowish and lacks luster, with symptoms of rapid breathing and wheezing, significantly reduced activity, decreased appetite, and enhanced stress response (easily agitated); ② Weight changes: The weight growth rate significantly decreases, and the weight at the end of the model period is significantly lower than that of the control group; ③ Pulmonary tissue pathological characteristics: Typical pulmonary emphysema and chronic bronchitis changes are presented, specifically manifested as rupture of alveolar walls, fusion of alveoli, expansion of alveolar cavities (significantly increased cross-sectional area of alveoli), thickening of airway walls, narrowing of airway cavities, increased mucus secretion in the airways, and extensive infiltration of inflammatory cells such as neutrophils and macrophages in the lung tissue and around the airways; ④ Pulmonary function indicators: Using a small animal pulmonary function detection system, the ventilation function indicators such as forced expiratory volume in 0.1 seconds (FEV0.1), forced expiratory volume in 0.05 seconds (FEV0.05), forced vital capacity (FVC), and vital capacity (VC) are significantly reduced. The establishment of the model is proved by the combination of the decline in lung function and the presence of pulmonary emphysema in the pathological changes.

(5) We greatly appreciate the reviewer’s professional and careful comment.

Antigen retrieval was successfully performed in all immunohistochemistry (IHC) experiments on model tissue sections. Briefly, all tissue sections were subjected to standardized heat-induced antigen retrieval using citrate buffer (pH 6.0) in a microwave oven at a consistent temperature and time, following widely accepted IHC protocols. The effectiveness of antigen retrieval was verified by clear, specific, and uniform positive staining in the targeted cells and structures, while no obvious nonspecific background staining was observed. All IHC procedures were performed in strict accordance with a unified, standardized protocol to ensure reproducibility and reliability of the results. We have added a brief description of the antigen retrieval method and verification in the revised manuscript to further clarify the details.

Furthermore, we have revised the legends of Figure 3D and Figure 6.

Reference：

[1]	LIU A, GAO X H, MAO Y, et al. Esketamine mitigates lung injury in COPD rat models under mechanical ventilation: An RNA-sequencing and bioinformatics analysis of serum exosome miRNA profiles [J]. Gene, 2025, 962: 149571.

[2]	LIU L, TANG Z, ZENG Q, et al. Transcriptomic Insights into Different Stimulation Intensity of Electroacupuncture in Treating COPD in Rat Models [J]. Journal of inflammation research, 2024, 17: 2873–87.

[3]	SHI M, XUE Q, XIE J, et al. Protective effect of Shenqi Wenfei Formula against lipopolysaccharide/cigarette smoke-induced COPD in Rat based on gut microbiota and network pharmacology analysis [J]. Frontiers in microbiology, 2024, 15: 1441015.

Reviewer #3: The study by Zhang et al. combines two publicly available COPD microarray datasets with multiple computational approaches, including WGCNA, immune cell analysis, Mendelian randomization, and machine-learning models, to identify diagnostic biomarkers for COPD. The authors highlight ITGB2 and HNRNPAB as key candidate genes and attempt to validate them using qPCR, IHC, and IF in cell line and mouse COPD models. This work is relevant because there is still a clear need for reliable biomarkers in COPD, and integrative computational approaches are increasingly used to study disease complexity. A major strength of the study is the broad computational pipeline that brings together multiple analytical methods and external validation datasets. However, the manuscript has major weaknesses that limit its scientific rigor and clarity, specially the mRNA/protein level discrepancy. Also, the writing style is unscientific and reflects conceptual misunderstandings, which makes the conclusions difficult to evaluate. In its current form, I cannot recommend this manuscript for publication unless these issues are carefully addressed. Please see my comments below.

1. Response to comment: A major weakness of the manuscript is the use of unscientific and conceptually incorrect language, along with a writing style that points readers to figures instead of explaining the results. For example, sentences such as “a gradient volcano plot (Fig. 2A) visualizes…” and “a heatmap (Fig. 2B) illustrates…” describe figures rather than findings and belong in figure legends, not the results section.

Also, the GSVA section uses incorrect terminology (e.g., “ITGB2 exhibited expression changes across pathways” or “KEGG pathway enrichment of ITGB2”). ITGB2 is a gene, and genes are not “expressed across pathways”, pathways do not have expression. These sound like conceptual errors rather than wording issues, and should be corrected throughout the manuscript.

Response: Special thanks to you for your exact and professional comments. We have revised the original text, described the results of the difference analysis, and completed the legend part in Figure 2. We have made it clear that ITGB2, as a gene, does not have "cross-pathway expression changes", and the pathway has no "expression" attribute. Such conceptual biases have been comprehensively corrected. We have carefully checked the relevant chapters of the full GSVA sentence by sentence to ensure that all terminology errors have been corrected and academic expressions have been standardized.

3. Response to comment: The manuscript reports “positive t-values” for GSVA results but never explains what these t-values represent, how they were calculated, or what comparison they correspond to. The t-statistics is not a p-value, and reporting “t-values” without p-values or FDR makes the results hard to interpret.

Response: Thank you sincerely for your meticulous review and highly constructive suggestions. In the GSVA analysis, the t-value represents the strength and direction of the association between gene expression and pathway activity. It is calculated through the GSVA algorithm based on the correlation statistics between pathway activity scores and gene expression levels. Positive t-values indicate a positive correlation between gene expression and pathway activity (high gene expression is accompanied by enhanced pathway activity). In the supplementary document, the P-values corresponding to each significant associated pathway have been refined to clarify the statistical reliability of the results. We have provided detailed explanations of the meaning of t-values, the calculation basis, and the corresponding comparisons (the correlation analysis between gene expression levels and GSVA pathway activity scores) in the main text and figure legends, ensuring that the results can be easily understood.4. Response to comment:

The reported AUC of 0.989 for a 12-gene COPD classifier trained on relatively small microarray datasets is unusually high for this kind of problem and raises concerns about overfitting. The cross-validation strategy and how the training and test sets were separated need to be explained much more clearly and evaluated more rigorously.

Response: We sincerely appreciate the reviewer’s rigorous and constructive concern regarding model performance, potential overfitting, and validation design. We fully agree that the high AUC value requires clearer explanation of training/test partitioning, cross-validation procedures, and generalization performance. We have thoroughly revised the Methods and Results sections to provide full transparency and rigorous validation details.

This study utilized the merged dataset of GSE37768 and GSE38974 for differential gene analysis, WGCNA, feature selection, and model construction. Three completely independent and non-overlapping datasets (GSE212331, GSE148004, GSE1650, etc.) were not involved in gene screening, feature selection, model training, and parameter tuning throughout the process. The training and test sets were strictly separated, ensuring that data leakage was avoided at the source and the evaluation of generalization ability was unbiased.

In the training set, 10-fold cross-validation (10-CV) was adopted, and nested validation was implemented: in each fold, feature selection and model fitting were independently completed within the training subset of the current fold; batch correction, standardization, gene filtering, and all preprocessing steps were only performed on the training subset within the fold; any information from the validation fold and the external test set did not flow into the model construction process. Nested cross-validation is a standard and rigorous approach in machine learning biomarker research to avoid overfitting.

We have added detailed descriptions of cross-validation, train–test partitioning, and external validation in the Methods (Section 2.6). These revisions fully clarify the model construction process and rigorously validate its generalization ability, thereby addressing concerns about overfitting.

We believe these changes provide complete transparency and rigorous evidence that the high AUC reflects genuine biological signal rather than overfitting.

5. Response to comment: The authors did not compare the 112 genes or the final 12-gene diagnostic panel with previously reported COPD transcriptomic signatures or genes linked with COPD (e.g., PMID-25265030 linking ITGB2 expression to COPD), making it difficult to assess what is truly novel versus a rediscovery of known signals.

Response: We sincerely appreciate the reviewer’s insightful and rigorous comment. We fully agree that comparing our gene sets with previously published COPD‑related transcriptomic signatures is essential to clarify the novelty and incremental contributions of this study. We have now systematically compared our 112 candidate genes and 12‑gene diagnostic panel with published COPD signatures and added detailed discussion in the revised manuscript to distinguish replicated known signals from novel findings.

Our study is the first to integrate 12‑algorithm ensemble machine learning (113 combinations), Mendelian randomization for causal inference, multi‑cohort independent validation, and experimental verification to identify and prioritize COPD diagnostic biomarkers, representing a methodological advancement over traditional differential expression‑only studies. This study focused on verifying HNRNPAB and ITGB2 through bioinformatics machine learning and Mendelian randomization methods.

We have compared our panel with reported COPD gene signatures. As the reviewer noted, ITGB2 has been associated with immune and inflammatory processes in COPD in previous studies[1]. This overlap supports the biological validity and reproducibility of our results. Notably, PMID: 25265030 focused on blood and sputum gene signatures for frequent COPD exacerbators and identified B3GNT, LAF4, and ARHGEF10 as predictive genes, which do not overlap with our 12‑gene panel. Our study focused on lung tissue‑based classification between COPD and healthy controls rather than exacerbation phenotype prediction, using a distinct study design and analytical framework.

Among the 12‑gene signature, HNRNPAB has not been previously reported as a diagnostic or causal biomarker for COPD and represents a novel finding of this study.

Liu and colleagues discovered that PDAP1, as a gene related to disulfide bonds, is involved in the expression of COPD through RNA sequencing and cell and animal experiments [2]. Most other genes in the panel (including CD300C, GNG13, LDB2, MCOLN3, NAIP, NOV, PDAP1) are also rarely highlighted in existing COPD diagnostic signatures.

Reference:

1. Singh D, Fox SM, Tal-Singer R, Bates S, Riley JH, Celli B. Altered gene expression in blood and sputum in COPD frequent exacerbators in the ECLIPSE cohort. PLoS One. 2014;9(9):e107381. Epub 2014/09/30. doi: 10.1371/journal.pone.0107381. PubMed PMID: 25265030.
2. Liu Y, Zhu T, Wang J, Cheng Y, Zeng Q, You Z, et al. Analysis of network expression and immune infiltration of disulfidptosis-related genes in chronic obstructive pulmonary disease. Immun Inflamm Dis. 2024;12(4):e1231. doi: 10.1002/iid3.1231. PubMed PMID: 38578019; PubMed Central PMCID: PMCQ3.

6. Response to comment: The FOXC1/NFIC–HNRNPAB axis is highlighted as a key regulatory mechanism, but the TF–gene network is built only from JASPAR motif predictions. There is no integration of expression correlations, no analysis of public ChIP‑seq data or promoter/enhancer motifs, and no functional assays (e.g., NFIC knockdown) to actually support these regulatory claims.

Response: We thank the reviewer for the critical suggestion. We have fully corrected the overstatement of the predicted TF–gene regulatory relationship, no longer claim the FOXC1/NFIC–HNRNPAB axis as a supported key mechanism, and clearly noted the preliminary nature of the in silico prediction. These revisions ensure the manuscript is rigorous, objective, and consistent with the actual experimental evidence.

7. Response to comment: The paper repeatedly labels ITGB2 and HNRNPAB as “key biomarkers” and frames them as a basis for personalized treatment, but all data are from mouse cells/mouse lungs and retrospective gene‑expression datasets. With no prospective human validation or demonstration that these markers improve on standard clinical measures, those clinical claims feel overstated and need to be toned down.

Response: We sincerely appreciate the reviewer’s rigorous and constructive comments. We fully agree that the clinical translation and validation scope of this study need to be accurately stated to avoid overstatement. We have systematically revised all overstated descriptions throughout the manuscript, clearly defined ITGB2 and HNRNPAB as candidate exploratory biomarkers (not clinically validated diagnostic markers), emphasized the limitations of murine and retrospective data, and clarified that these findings provide a preliminary theoretical basis rather than mature clinical application evidence.

8. Response to comment: The discussion section is excessively long and repetitive, with too much speculation that goes beyond the data presented. It should be shortened and focused on the main findings that are directly supported by the results and key limitations, with speculative sections reduced or removed to improve clarity and impact

Response: Thank you for your reminder. We have reduced the speculative part in the discussion to truly reflect the research results.

Reviewer #4: In this manuscript, Zhang et al. integrate multiple bioinformatics and machine-learning approaches to identify potential COPD biomarkers from gene expression data derived from multiple datasets. The study combines network-based analyses (co-expression and regulatory networks), pathway-level analyses, Mendelian randomization, and machine-learning methods to prioritize candidate biomarkers, followed by in vivo validation. Among the candidates, ITGB2 and HNRNPAB were validated by PCR, IHC, and IF experiments in a mouse model.
The manuscript is generally well written, and the analyses are appropriately conducted. I believe that minor revisions would further improve the clarity, consistency, and completeness of the work prior to publication.

1. Response to comment:

1. Lines 123–124:
The sentence ending at line 124 appears to be incomplete, as it ends with a comma. Please revise the sentence to ensure it is grammatically complete. Thank you for your guidance. We have now completed the sentence.
2. Figure 3D:
Figure 3D is not explicitly described or referenced in the main text.
Please add a brief explanation in the Results section to clarify the purpose and interpretation of this panel. We sincerely appreciate the reviewer’s rigorous and constructive comments. We explained Figure 3D. In the turquoise module, the correlation between the module member value (MM) and the gene significance (GS). The MM (correlation between the gene and the module feature gene) and GS (correlation between the gene and the treatment phenotype) of each gene were visualized through a scatter plot. The results showed that there was a significant positive correlation between the two (correlation coefficient cor=0.5, P=7.1×10⁻⁴⁹). This strong correlation indicates that genes with higher connectivity in the turquoise module also have stronger significance with the treatment phenotype, suggesting that this module has a high biological correlation with the treatment response. The above results support prioritizing the genes with high connectivity in the turquoise module as candidate biomarkers or functional regulatory factors for analyzing the treatment effect.

Also, the figure legend for Fig. 3D appears to be identical to that of Fig. 3B. Please revise the legend to accurately describe the content of Fig. 3D. Thank you for your reminder. We have made modifications to the legend.
3. Figure 4 legends:
The figure legends for Fig. 4B and Fig. 4C appear to be reversed. Please confirm and correct the legends to match the corresponding panels. Thank you for your suggestion. We have already modified the legend.
4. Lines 486–488:
Two consecutive sentences begin with “On the other hand.” I recommend revising this part to improve readability and sentence flow. Thank you for your reminder. We have revised the original text to enhance the readability of the sentences.
5. Reproducibility and code availability:
For clarity and reproducibility, it may be helpful for the authors to indicate whether the analysis code used in this study is available, for example in a public repository or upon reasonable request.

Response: We thank the reviewer for the critical suggestion. We have already disclosed the author information in the data availability section and are ready to answer readers' inquiries about the code application.
