# Supplementary material for: Identifying and validating ITGB2 and HNRNPAB as diagnostic biomarkers in chronic obstructive pulmonary disease using bioinformatics and Integrated Machine Learning Methods
Source: PLoS One. 2026 May 21;21(5):e0349338. doi: 10.1371/journal.pone.0349338 (PMC13193535; doi:10.1371/journal.pone.0349338)
Supplement: Attachment — Submitted filename: Response_to_Reviewers_auresp_3.docx [file pone.0349338.s008.docx]

List of Responses

Dear Tomasz W. Kaminski and Reviewer:

Thank you for your letter and for the reviewers’ comments concerning our manuscript entitled “Identifying and Validating ITGB2 and HNRNPAB as diagnostic biomarkers in chronic obstructive pulmonary disease Using Bioinformatics and Integrated Machine Learning Methods”. (Manuscript Number: PONE-D-25-52871). Those comments are all valuable and very helpful for revising and improving our paper, as well as the important guiding significance to our research. We have studied comments carefully and have made corrections which we hope meet with approval. Revised portions are marked with a yellow background in the revised version. The main corrections in the paper and the responds to the reviewer’s comments are as flowing:

Responses to reviewer’s comments: (original comments by reviewers are in blue color).

Key points to address:

a) Methodological justification

Please provide a clearer rationale for key analytical choices, particularly:

-the use of the IVW method as the primary Mendelian randomization approach,

-the selection and source of SNPs used in the analysis.

Thank you for your review. In the Mendelian randomization method guide [1], IVW is a meta-analysis method that takes the weighted average of all IV effects and contributes more to high-precision SNPS. The research of Stephen Burgess et al. [2] showed that the IVW method is most efficient when all instrumental variables are valid. In Mendelian randomization studies, IVW is the most mainstream and core method for estimating the causal relationship between exposure and outcome [3]. Therefore, we apply the IVW method as the main approach.

SNPs were selected from genome-wide significant pQTLs in the deCODE database, pruned for linkage disequilibrium, filtered for instrument strength, and harmonized with FinnGen outcome data, ensuring valid instrumental variables for MR analysis. We have provided detailed explanations in Section 2.9 of the manuscript. The revisions can be found on page 9 of the manuscript, please refer to the yellow-marked part on page 9 of the original manuscript, lines 189-192.

Reference:

1.	Nguyen K, Mitchell BD. A Guide to Understanding Mendelian Randomization Studies. Arthritis Care Res (Hoboken). 2024;76(11):1451-60. doi: 10.1002/acr.25400. PubMed PMID: 39030941; PubMed Central PMCID: PMCQ2.

2.	Burgess S, Foley CN, Allara E, Staley JR, Howson JMM. A robust and efficient method for Mendelian randomization with hundreds of genetic variants. Nat Commun. 2020;11(1):376. doi: 10.1038/s41467-019-14156-4. PubMed PMID: 31953392; PubMed Central PMCID: PMCQ1.

3.	Mounier N, Kutalik Z. Bias correction for inverse variance weighting Mendelian randomization. Genet Epidemiol. 2023;47(4):314-31. doi: 10.1002/gepi.22522. PubMed PMID: 37036286; PubMed Central PMCID: PMCQ1.

b) TF-gene regulatory network

Please clarify the basis of this network (e.g., binding site prediction vs co-expression vs functional inference) and discuss its limitations.

We thank the reviewer for this important comment. We agree that the basis of the TF-gene regulatory network should be described more explicitly.

In the present study, the TF-gene regulatory network was constructed using the JASPAR motif database through the NetworkAnalyst 3.0 platform, as already indicated in the Methods section of the manuscript. Therefore, this network is a motif-based/prediction-based regulatory network, built from curated transcription factor binding profiles rather than from co-expression analysis or direct experimental evidence such as ChIP-seq. Specifically, JASPAR provides manually curated transcription factor binding profiles represented as position frequency matrices (PFMs), which can be used to scan DNA sequences and infer putative TF-binding events, while NetworkAnalyst offers a framework to map these predicted TF-gene relationships into a regulatory network.

We have now clarified in the revised manuscript that the TF-gene network should be interpreted as a hypothesis-generating computational network. It suggests potential upstream regulators of the identified COPD-related biomarkers, but it does not demonstrate direct physical TF binding or definitive transcriptional regulation in COPD tissue. We also added a paragraph discussing the limitations of this analysis. Accordingly, we have revised both the Methods and Discussion sections to better define the analytical basis of the network and to discuss its limitations in a more balanced manner. The revisions can be found on page 21 of the manuscript, please refer to the yellow-marked part on page 21 of the original manuscript, lines 464-466 and 470-472.

c) Gene prioritization

The focus on ITGB2 and HNRNPAB requires stronger justification. Please clearly explain why these genes were selected over other candidates.

Thank you for your reminder. We obtained 12 key COPD biomarkers through bioinformatics and integrated machine learning methods, and finally identified ITGB2 and HNRNPAB by Mendelian randomization. It is well known that the Mendelian randomization method uses genetic variations (such as SNPS) as instrumental variables to simulate "natural randomized controlled trials", and thereby infer the causal relationship between biomarkers and diseases. The molecules obtained from the aforementioned bioinformatics analysis (differential analysis, WGCNA, and integrated machine learning methods) were only correlated with COPD. The final Mendeldahl randomization revealed that ITGB2 and HNRNPAB were causal to COPD, significantly enhancing the biological credibility of the screening. This was confirmed in subsequent animal and cell experiments.

d) Coonsistency of interpretation

The reference to “treatment response” in the WGCNA analysis is inconsistent with the study design (COPD vs control) and should be revised.

Thank you for your review. We have made modifications to the text and image annotations. The revisions can be found on page 14 of the manuscript, please refer to the yellow-marked part on page 14 of the original manuscript, lines 311-313.

e) Methods presentation

Sections describing HE staining, immunohistochemistry, and immunofluorescence should be rewritten in a concise scientific format rather than step-by-step protocol style.

Thank you for your valuable feedback. Your careful review has provided important guidance for the writing style of this section. We have thoroughly revised the sections on HE staining, immunohistochemistry, and immunofluorescence to ensure they adhere to the standards of a scientific abstract. The revisions can be found on pages 12 and 13 of the manuscript, please refer to the sections marked in yellow on pages 12 and 13 of the manuscript, specifically lines 266–270, 282–285, and 287–291.

f) Technical and formatting issues

Please correct formatting errors in Section 2.13. Carefully proofread the manuscript for typographical errors, gene name consistency, and figure legend accuracy.

We have revised the original text to ensure the consistency of the gene names and legends. The revisions can be found on pages 12 and 13 of the manuscript, please refer to the yellow-marked part on page 12 of the original manuscript, lines 260-261.

g) Positioning of conclusions

The proposed gene signature should be presented more cautiously as exploratory, given the nature of the data and analytical pipeline.

Thank you for your review. We screened the two molecules ITGB2 and HNRNPAB through bioinformatics and integrated machine learning methods, and detected the expression of these two molecules in animal and cell experiments, making them exploratory biomarkers for COPD, with the aim of testing and intervening in future population cohorts. Therefore, our conclusion is that this is an exploratory discovery.

The revisions can be found on page 3 of the manuscript, please refer to the yellow-marked part on page 3 of the original manuscript, lines 48-50.

Reviewer #3: The revised manuscript has improved significantly. I am still somewhat unconvinced by the mRNA–protein discrepancy, but this limitation is now acknowledged and possibilities are discussed. Overall the language is acceptable, key claims have been toned down, limitations are recognized, and the conceptual errors have been corrected. I therefore recommend the manuscript for acceptance.

Response: Thank you for your reminder.

Reviewer #4: I appreciate that the authors have addressed my previous comments in the revised manuscript. On reviewing the revised version, however, I have identified several additional issues that should be addressed to further improve the clarity and consistency of the manuscript.

1. Response to comment:

1. Methods: Section 2.13 Lung Function Test:

There appears to be a formatting issue in this section, where the content is inserted between the text "Methods for Lung Function Testing". I recommend carefully proofreading the manuscript prior to submission to avoid such issues.

Thank you for your guidance. We have revised the original text. The revisions can be found on pages 12 and 13 of the manuscript, please refer to the yellow-marked part on page 12 of the original manuscript, lines 260-261.
2. HE staining, Immunohistochemistry and Immunofluorescence methods:

The description of methods is currently presented in a highly step-by-step manner, for example repeated use of imperative expressions such as "Place the sections ...". While detailed protocols are useful, this style reads more like a protocol than a methods section appropriate for a research article.

Thank you for your suggestion. We have made the revisions you suggested. The revised content can be found on pages 12 and 13 of the manuscript, highlighted in yellow, specifically lines 266 through 270.

3. Results: Section 3.2 (WGCNA analysis):

The interpretation of the WGCNA results may require clarification. The statement "confirming its critical role in the treatment response" appears inconsistent with the study design, which is based on COPD versus control samples rather than treatment conditions. It is unclear whether any treatment-related variables were included in the analysis. This may be due to the labeling (e.g., "Treat" vs "Ct") used in the dataset, which could potentially lead to confusion in interpretation. I recommend revising these labels and the associated text to ensure that the interpretation is consistent with the analyzed dataset conditions.

Thank you for your suggestion. We have made modifications to the text and image annotations. The revisions can be found on page 14 of the manuscript, please refer to the yellow-marked part on page 14 of the original manuscript, lines 311-313.
