# Supplementary material for: Identifying and validating ITGB2 and HNRNPAB as diagnostic biomarkers in chronic obstructive pulmonary disease using bioinformatics and Integrated Machine Learning Methods
Source: PLoS One. 2026 May 21;21(5):e0349338. doi: 10.1371/journal.pone.0349338 (PMC13193535; doi:10.1371/journal.pone.0349338)
Supplement: Attachment — Submitted filename: Response_to_Reviewers_auresp_4.docx [file pone.0349338.s009.docx]

List of Responses

Dear Tomasz W. Kaminski:

Thank you for your letter and for the reviewers’ comments concerning our manuscript entitled “Identifying and Validating ITGB2 and HNRNPAB as diagnostic biomarkers in chronic obstructive pulmonary disease Using Bioinformatics and Integrated Machine Learning Methods”. (Manuscript Number: PONE-D-25-52871). Those comments are all valuable and very helpful for revising and improving our paper, as well as the important guiding significance to our research. We have studied comments carefully and have made corrections which we hope meet with approval. Revised portions are marked with a yellow background in the revised version. The main corrections in the paper and the responds to the reviewer’s comments are as flowing:

Responses to reviewer’s comments: (original comments by reviewers are in blue color).

a) In the Results section (Sections 3.9-3.10), you report increased mRNA but decreased protein levels for ITGB2 and HNRNPAB. Please acknowledge this discrepancy already in the Results (not only in the Discussion) to guide the reader.

Thank you for this suggestion. We have now added a sentence at the end of Section 3.9 (Page 19, lines 419-422) to explicitly acknowledge the mRNA-protein discrepancy in the Results section. We also retained the more detailed mechanistic discussion in the Discussion section.

b) Your validation includes both MLE-12 cells and whole lung tissue. Please briefly clarify which cell types are likely contributing to the observed signals in tissue-based analyses and note potential differences between epithelial cells and mixed tissue samples.

We appreciate this comment. We have added a clarifying statement in Section 3.9 (Page 19, lines 412-415) indicating that tissue IHC/IF signals originate from mixed cell populations (epithelial, immune, stromal cells), while MLE-12 cells are a pure epithelial line. This highlights the potential differences between tissue-based and cell line results.

c) The reported AUC values (up to 0.989) are very high. Please add a brief comment in the Discussion acknowledging the potential for overfitting or dataset-specific bias, even though external validation cohorts were used.

Thank you for your review. We have already explained in the discussion that an excessively high AUC of the predictive model integrating machine learning may be related to data overfitting or specific bias of the sample dataset. The revisions can be found on page 24 of the manuscript, please refer to the yellow-marked part on page 24 of the original manuscript, lines 559-560.
